# Evaluation of Total Antioxidant Activity and Oxidative Stress in Seminal Plasma from Dogs Supplemented with Fish Oil and Vitamin E 

**DOI:** 10.22074/IJFS.2021.6226

**Published:** 2021-01-27

**Authors:** Analía Risso, Francisco Javier Pellegrino, Yanina Corrada, Guillermo Schinella

**Affiliations:** 1 National Council of Research and Technology (CONICET), Buenos Aires, Argentina; 2 Institute of Veterinary Genetics (IGEVET, CONICET LA PLATA), School of Veterinary Sciences (FCV), National University of La Plata (UNLP), La Plata, Buenos Aires, Argentina; 3 School of Medical Sciences (FCM, UNLP); Institute of Health Sciences (National University Arturo Jauretche-CICPBA), Buenos Aires, Argentina

**Keywords:** Antioxidants, Dog, Fish Oil, Oxidative Stress, Vitamin E

## Abstract

**Background:**

We evaluated the effect of fish oil (FO) and FO in addition to vitamin E (VE) supplementation on total antioxidant activity of dog seminal plasma, and further assessed oxidative stress. Additionally, we measured the effect of this supplementation on hematological parameters and serum biochemistry.

**Materials and Methods:**

In this experimental study, six male dogs were assigned to one of the following three groups for a period of 60 days using a replicated 3×3 Latin square design: control (CG), FO (FOG) and FO in addition to VE (FOEG). On days 0 and 60 of the trial, semen and blood samples were obtained. 2,2V-azino-bis (3-ethylbenzo- thiazoline-6-sulfonate) (ABTS) and ferric reducing antioxidant power (FRAP) assays were used to determine total antioxidant activity. Oxidative stress was determined by measuring total sulfhydryl group (T-SH).

**Results:**

Dogs supplemented with FO alone had a lower total antioxidant activity in seminal plasma (ABTS: -59.86% vs. CG and -57.3% vs. FOEG; and FRAP: -37.3% vs. CG and -40.5% vs. FOEG), and higher oxidative stress (T-SH: +53.0% vs. CG and+60.2% vs. FOEG) compared with the other two groups (P<0.05). Serum triglyceride (TG) concentration de- creased in FOG and FOEG compared with CG, on day 60 (P<0.01).

**Conclusion:**

We concluded that total antioxidant activitydecreased and oxidative stress increased in seminal plasma of dogs after FO supplementation for 60 days.

## Introduction

Omega-3 polyunsaturated fatty acids (ɷ-3 PUFA) are lipids with important biological functions that are found in fish oils (FO). They primarily include eicosapentaenoic acid (EPA, C20:5 ɷ-3) and docosahexaenoic acid (DHA, C22:6 ɷ-3). In dogs, FO supplementation improved sperm parameters and increased the percentage of EPA and totalɷ-3 PUFA in semen (1). However, PUFA such as EPA, DHA and arachidonic acid (AA, C20:4 ɷ-6) have a high risk of oxidation related to the number of double bonds in their molecules (2). PUFA oxidation can increase oxidative stress in cells. It been studied that the relationship among dietary fatty acid supplementation, sperm PUFA composition and total antioxidant activity, affects semen quality in animals’ species (3). In order to balance high dietary ɷ-3 PUFA intake, adequate antioxidant concentrations are needed. 

Antioxidants can be endogenous or exogenous; the latter can be obtained from the diet or dietary supplements. In this context, vitamin E (VE) is among non-enzymatic antioxidants in the body and plays an important role in protecting many different cells and organs against oxidative stress. Reactive oxygen species (ROS) are by-products of cellular metabolism which regulate physiological processes at low-to-moderate levels. However, antioxidant defenses may be inadequate to inactivate ROS, thereby, oxidative stress occurs and damages nucleic acids, lipids and proteins (4). Animals develop enzymatic and non-enzymatic antioxidant systems to reduce or prevent potential oxidative damage. In seminal plasma of dogs, antioxidant enzymes are produced by the testis, epididymis and accessory reproductive organs (5). Although a small amount of ROS is needed for capacitation, hyper activation, motility and acrosome reaction of the sperm as well as fertilization (6), the impactof ROS overproduction on antioxidant defense mechanisms may result in oxidative stress (7).

We previously observed that sperm lipid peroxidation in dogs decreased with FO alone or FO plus VE supplementation, indicating a potentially protective effect of ɷ-3 PUFAand VE (8). However, antioxidant activity and oxidative stress in seminal plasma of dogs supplemented with FO alone or FO plus VE, have not been reported thus far. Consequently, here, we evaluated such effects for the first time in dog seminal plasma, and additionally determined the effect of the same supplementations on hematological and biochemical parameters. We hypothesized that FO supplementation would produce lower total antioxidant activity and higher oxidative stressthan FO in addition to VE, in dog seminal plasma.

## Materials and Methods

### Animals and treatments

In this experimental study, the Institutional Animal Care and Use Committee of the School of Veterinary Sciences, National University of La Plata, Buenos Aires, Argentina approved the study protocol (No. T34-1-13). Healthy male fertile dogs (age range 2-5 yr. and body weight [BW] range 20-30 kg), were used. Body condition score was 3 on a 5-point scoring scale (9). Clinical examination data (clinical record and physical exam), routine blood and biochemical test results and semen parameters were used to assess health status. Dogs were adapted to a standard commercial food (control diet) 15 days before the study. The nutrient composition of the commercial food described on a dry matter basis (%) was as follows: 3.7 metabolizable energy (kcal/g), 30.04 crude protein, 15.02 fat, 1.72 neutral detergent fiber, 7.83 ash, 1.50 calcium, 1.07 phosphorus, 0.048 mineral and vitamin mixture with: 12.39 VE, 0.20 vitamin K, 0.82 vitamin B1, 0.82 vitamin B2, 0.82 vitamin B6, 0.0004 vitamin B12, 0.12 folic acid, 0.10 nicotinic acid, 2.06 calcium pantothenate, 0.02 biotin, 82.1 choline, 0.01 copper, 0.01 iron, 0.02 zinc, 0.003 iodine, 0.01 manganese and 0.0002 selenium). Main ingredients of the extruded pelletwere: chicken meal, wheat, beef meal, rice, chicken oil, micronized soybean meal, gluten meal, corn, beet-root pulp, hydrolyzed chicken protein, fish oil, beer yeast, zeolite, salt, vitamin C, inulin, Yucca schidigera extract, antioxidants, potassium sorbate, yeast walls, yeast nucleotides, sodium hexametaphosphate, methionine and lysine.

Daily dietary intake was controlled and calculated according to the maintenance energetic requirements [MER=132 kcal×kg metabolic BW (BW0.75)] (10). Dogs had free access to water.

We conducted a randomized controlled trial using the random list generated by the computer soft ware and a replicated 3×3 Latin square design. Six crossbreed dogs were assigned to one of the following three groups: i. Control (CG; daily intake of the control diet), ii. FO (FOG; daily intake of the control diet plus a capsule containing the FO supplement dose of 54 mg FO/kg BW0.75), and iii. FO+VE (FOEG; daily intake of FOG plus 400 mg VE (8)). Individual FO dose was calculated as reported by Risso et al. (1). The FO capsule was administered daily with the first meal. Each experimental stage lasted 60 days. Finally, a 60-day washout among periods of treatments was included to avoid the carry-over effect of supplementation; during this period dogs were only given the control diet. This washout time was checked and evaluated in a previous study (8).

Dog owners gave written informed consent and committed to complying with the study protocol, i.e. feeding their dogs with the CG, FO or FOG diets. Furthermore, dogs were taken to the School of Veterinary Sciences everyweek during the experimental period to register their food intake and body weight. All evaluations were carried out by masked independent investigators.

### Seminal plasma sample collection

Dogs were trained for semen collection by manual stimulation before starting the study. After sample collectionon days 0 and 60,1-ml aliquotsfrom each sample were centrifuged at 800g for 10 minutes to separate sperm from seminal plasma. After centrifugation, seminal plasma was snap-frozen and stored at -83°C until use (11).

### Total antioxidant activity

Two assays were used to determine total antioxidant activityin dog seminal plasma: 2,2V-azinobis (3-ethylbenzothiazoline 6-sulfonate) (ABTS) and ferric reducing ability of plasma (FRAP), as described by Katalinic et al. (12), with some modifications. An ascorbic acid curve was established and the results were expressed as μg of ascorbic acid equivalents per ml. All measurements were performed in duplicate.

### Oxidative stress

Oxidative stress in seminal plasma was determined by measuring total sulfhydryl groups (T-SH) using Ellman´s method with some modifications (13). A reduced glutathione (GSH) calibration curve was constructed; results were expressed as micrograms of GSH equivalents per milligrams of protein. All measurements were made in duplicate. Total seminal plasma protein concentration was determined using Bradford´s assay (14).

### Blood samples

Venous blood samples for hematological and biochemical analyses were collected on days 0 and 60. Dogs were not fed or given waterfor at least 12 hours before the blood collection. Whole blood (5-ml) was drawn through venipuncture of the cephalic vein using a 21G needle and transferred to 1-mlEDTA tubes and 4-ml tubes without additives. Serum was separated by centrifugation at 1400 g for 5 minutes immediately after collection and then, transferred to another tube for biochemical analysis. Samples collected in tubes with or without EDTA were stored at 4°C for subsequent hematological and biochemical analyses within 6 hours after blood withdrawal.

### Hematological and biochemical analyses

Hematological analysis was performed using an automated cell counter (Sysmex-KX-21) for the following variables: 

erythrocytes, leukocytes, platelets, hemoglobin, hematocrit, mean corpuscular volume, mean corpuscular hemoglobin and mean corpuscular hemoglobin concentration.

Serum biochemical analysis of total solids, total protein, albumin, globulins, glucose, alanine aminotransferase, urea, creatinine and triglyceride (TG) concentrations was performed on an Intelligent Clinical Chemistry Analyzer (INCAA, DICONEX, Argentina).

### Standard commercial food analysis

Once pooled, standard commercial food was analyzed to determine first: dry matter (at 80ºC for 48 hours), and then, neutral detergent fiberfraction (NDF, Ankom 200 Fiber Analyzer, ANKOM Technology, Fairport, NY), crude protein fraction (CP, KjeldahlN x 6.25), lipids fraction (in the ether extract, XT101 ANKOM Technology Method 2) and finally ash (15).

### Fatty acid composition of the standard commercial food and fish oil

For lipid extraction, samples of the standard commercial food and FO were studied as described by Folch et al. (16). Fatty acid composition was measured using gas chromatography working with a 30-mm capillary column [Omega Wax 250; Supelco, Bellefonte, PA, USA (Table 1)].

**Table 1:** Fatty acid content (%) of the standard commercial food and the fish-oil supplemented one


Fatty acid	Standard food	Fish oil

Tetradecanoic (14:0)	0.8	1.8
Palmitic (16:0)	20.9	24.0
Palmitoleic (16:1)	6.0	10.1
Stearic (18:0)	6.3	3.4
Oleic (18:1 ɷ-9)	27.9	22.3
Vaccenic (18:1 ɷ-7)	-	1.3
Linoleic (18:2 ɷ-6)	27.8	2.0
Gamma-linolenic (18:3 ɷ-6)	1.4	-
Alpha-linolenic (18:3 ɷ-3)	3.4	2.1
Eicosenoic (20:1 ɷ-9)	0.1	2.5
Dihomo-gamma-linolenic (20:3 ɷ-6)	0.2	-
Eicosatrienoic (20:3 ɷ-3)	0.4	-
Arachidonic (20:4 ɷ-6)	1.4	2.0
Eicosatetraenoic (20:4 ɷ-3)	-	0.8
Eicosapentaenoic (20:5 ɷ-3)	0.1	9.6
Docosapentaenoic (22:5 ɷ-6)	0.2	-
Docosapentaenoic (22:5 ɷ-3)	0.2	1.2
Docosahexaenoic (22:6 ɷ-3)	0.2	16.9
Σ Saturated fatty acids	28.0	29.2
Σ Monounsaturated fatty acids	34.0	36.2
Σ Polyunsaturated fatty acids	35.3	34.6
Σ ɷ-6	31.3	4.0
Σ ɷ-3	4.3	30.6


ɷ-9; Omega 9, ɷ-7; Omega 7, ɷ-6; Omega 6, and ɷ-3; Omega 3.

### Statistical analysis 

Areplicated 3×3 Latin square was considered. All the squares had two dogs that received three treatments. Data were registered in an Excel® database (Microsoft, USA) and processed using GraphPad Prism 4 for Microsoft Windows® (GraphPad Software, USA). Considering that a previously checked wash out period between treatments was included (8), data of antioxidant activity and oxidative stress were analyzed using oneway ANOVA followed by Tukey-Kramer test for multiple comparisons. Results of total antioxidant activity and oxidative stress assays areexpressed as means ± standard deviation (SD). 

Data of hematological and biochemical parameters were analyzed by SAS PROC MIXED (version 9.4; SAS Institute Inc., Cary, NC, USA), with repeated measurements. The model contained within the random effect of dogs, the period of replicated Latin square design, the fixed effect of time (0 and 60 days), treatment (CG vs. FOG vs. FOEG), replication and time by treatment interaction. The time points with significant differences in time by treatment interaction were detected with the slice option of SAS. Significance was set at P<0.05. Data of hematological and biochemical analyses areexpressed as least square means (LSM) with standard error of means (SEM).

## Results

The average age of dogs was 3.1 ± 1.4 years and they weighed 26.3 ± 2.1 kg.

### Total antioxidant activity

Total antioxidant activity of dog seminal plasma did not differamongthe groups on day 0 (P>0.10). Conversely, it was lower in FOG on day 60, as shown by lower ABTS radical discoloration (CG, 1.52 ± 0.80 Eq µg ascorbic acid/ml; FOG, 0.61 ± 0.44 Eq µg ascorbic acid/ml; and FOEG, 1.43, ± 0.69 Eq µg ascorbic acid/ml) and lower FRAP (CG, 1.66 ± 0.63 Eq µg ascorbic acid/ml; FOG, 1.04 ± 0.41 Eq µg ascorbic acid/ml; and FOEG, 1.75 ± 0.79 Eq µg ascorbic acid/ml) compared toCG and FOEG (treatment effect, P<0.05, Figes.1, 2).

**Fig.1:**
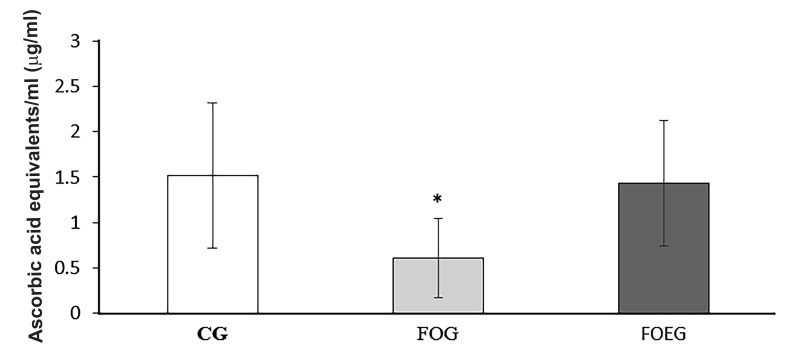
Total antioxidant activity in dog seminal plasma determined by 2,2V-azinobis (3-ethylbenzothiazoline 6-sulfonate) (ABTS) assay in the control (CG, n=6), fish oil (FOG, n=6) and fish oil in addition to vitamin E group (FOEG, n=6) on day 60. *; Treatment effect, P<0.05.

**Table 2:** Hematological and biochemical parameters in samples from dogs assigned to the control (CG, n=6), fish oil (FOG, n=6) and fish oil in addition to vitamin E group (FOEG, n=6), on days 0 and 60


Blood parameters	Day 0				Day 60			
	CG	FOG	FOEG		CG	FOG	FOEG	

	LSM	LSM	LSM	SEM	LSM	LSM	LSM	SEM
Erythrocytes (×10^6^/µl)	7.07	7.35	6.27	0.46	7.27	7.15	7.02	0.40
Leukocytes (×10^3^/µl)	10.29	11.11	7.98	1.48	12.43	11.83	10.43	1.26
Platelets (×10^5^/µl)	2.86	2.61	2.65	0.76	3.82	1.97	2.78	0.74
Hemoglobin (%)	15.72	15.76	15.21	0.70	16.49	17.18	17.67	0.65
Hematocrit (%)	46.53	48.37	47.48	2.00	45.73	47.55	48.27	1.75
MCV (fl)	67.88	67.92	70.28	0.82	68.80	67.91	68.13	0.70
MCH (pg)	24.11	24.30	24.75	0.45	24.22	23.95	23.65	0.38
MCHC (%)	35.66	35.74	35.82	0.61	35.10	35.28	34.19	0.56
Total solids (g/dl)	6.52	6.34	6.86	0.39	6.12	6.22	6.58	0.35
Total protein (g/dl)	5.97	6.39	6.30	0.35	6.05	6.31	6.27	0.28
Albumin (g/dl)	3.08	2.86	2.35	0.25	3.35	2.85	2.60	0.19
Globulin (g/dl)	3.00	3.21	3.72	0.37	2.72	3.22	3.48	0.31
Glucose (g/l)	0.81	0.69	0.83	0.10	0.76	0.87	0.82	0.12
ALT (U/l)	32.80	40.24	47.37	8.69	30.29	44.92	34.29	9.29
Urea (g/l)	0.59	0.56	0.70	0.10	0.58	0.61	0.66	0.11
Creatinine (mg/dl)	1.01	0.97	1.18	0.35	0.95	0.90	1.38	0.38
Triglycerides (g/l)	0.88	1.04	1.07	0.16	1.15	0.75^*^	0.56^*^	0.15


LSM; Least square mean, SEM; Standard error of means, MCV; Mean corpuscular volume, MCH; Mean corpuscular hemoglobin, MCHC; Mean corpuscular hemoglobin concentration, ALT, Alanine aminotransferase, and *; Treatment × time interaction, P<0.01. The remaining parameters were not significantly different (P>0.05).

**Fig.2:**
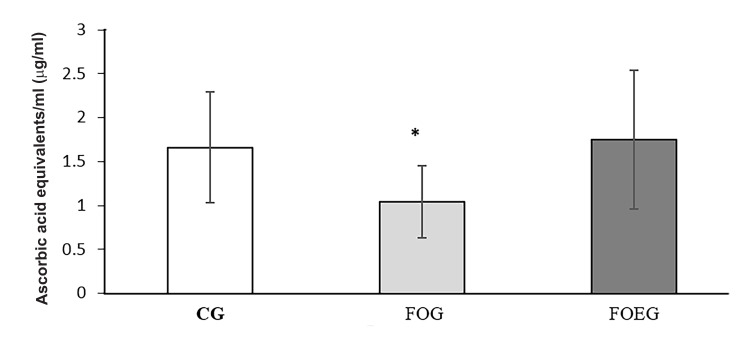
Total antioxidant activity in dog seminal plasma determined by ferric reducing ability of plasma (FRAP) assay in the control (CG, n=6), fish oil (FOG, n=6) and fish oil in addition to vitamin E group (FOEG, n=6) on day 60. Values are means and standard deviation. *; Treatment effect, P<0.05.

### Oxidative stress

**Fig.3:**
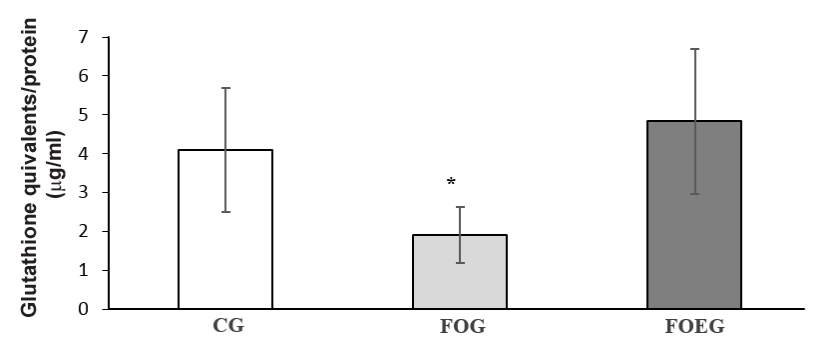
Oxidative stress determined in terms of total sulfhydryl groups (TSH) in seminal plasma of dogs in the control (CG, n=6), fish oil (FOG, n=6) and fish oil in addition to vitamin E group (FOEG, n=6), on day 60. Values are means and standard deviation. *; Treatment effect, P<0.05.

In seminal plasma, T-SH content assessment showed that oxidative stress was higher in FOG (1.92 ± 0.72 Eq µg glutathione/mg protein) compared to CG (4.09 ± 1.59 Eq µg glutathione/mg) and FOEG (4.83 ± 1.86 Eq µg GSH /mg) after 60-day supplementation (treatment effect, P<0.05, Fig .3).

### Hematological and biochemical parameters

Serum TG concentration decreased in FOG and FOEG compared to CG on day 60 (treatment×time interaction, P<0.01). No effect for FO alone or FO plus VE supplementation was observed on the other hematological and biochemical parameters (P>0.05, Table 2).

## Discussion

In this study, the effect of FO alone or FO with VE supplementation on total antioxidant activity and oxidative stress was assessed in seminal plasma of dogs. Additionally, the effect of both supplements on hematological and biochemical parameters was evaluated. In accordance with our hypothesis, FO supplementation decreased total antioxidant activity after 60 days, while increasing oxidative stress. 

Seminal plasma is composed of proteins, amino acids, enzymes, carbohydrates, lipids, major minerals and trace elements (17). The lipid fraction only contains 2.5% fatty acids, from which, 85% are saturated fatty acids and 15% are unsaturated fatty acids (13.3% monounsaturated fatty acids and1.7% PUFA) (18).

In previous studies on dietary FO supplementgiven to dogs, we observed an increase in AA, EPA and total ɷ-3 PUFA concentrations in semen. Although in the present study we did not evaluate PUFA composition in seminal plasma, its concentration could have been affected by the ɷ-3PUFA content in the FO-supplemented diet (1, 8). Such increases in seminal plasma ɷ-3 PUFA concentrations could be the result of an imbalance between the antioxidant and oxidant systems, with concomitant decreases in total antioxidant activity and increases in oxidative stress.

VE has been shown to directly neutralize superoxide anion, hydrogen peroxide and hydroxyl radical (19). In this sense, Domosławska et al. (17) reported that supplementation with selenium and VE for 60 days enhanced the antioxidant status of sperm in dogs. In the present study, the higher VE content in FOEG could have maintained total antioxidant activity/oxidative stress balance in seminal plasma.

Another report showed the effects of PUFA consumption on oxidative status in dogs. Dogs received PUFA-rich diets with the proposed of modifying oxidative stress markers in blood; however, the concentration used was not enough to cause an imbalance between the generation and elimination of reactive species by the antioxidant defense systems of dogs. In the cited report, treatments contained 20% acid hydrolyzed fatand 60% PUFA inDHA-enriched soybean oil, and 18% PUFA in bovine tallow (20). In agreement with those results, the PUFA content in the diets of the present study met the maintenance requirements for dogs recommended by the National Research Council 2006 (10). 

The National Research Council 2006 (10) cites that diets with more than 50% fatty acid dry matter, alter the antioxidant system. Although in the present study, oxidative stress was not determined directly in blood samples, the evaluated hematological and biochemical parameters (i.e. albumin and total protein) did not show differences among the study groups. On the other hand, the results obtained in seminal plasma may reflect a different stage from that observed in blood results. Consequently, simultaneous evaluation of total antioxidant activity and oxidative stress in blood and seminal plasma, would be useful for a better interpretation of results.

Regarding the lower serum TG concentrations found in FOG and FOEG,our results support the already reported role of ɷ-3 PUFA in lipid metabolism (21). Thus, the FO dose currently used could be consideredsafe for treatment of hyperglycemia in dogs.

## Conclusion

We observed that the decreased antioxidant activity and increased oxidative stress found in seminal plasma of dogs supplemented with FO, would be related to an increased risk of oxidative stress due to higher amounts of PUFA in the diet. Supplementation with FO in addition to VE would exert a protective role against ROS. Further studies are needed toclarify the mechanisms responsible for such total antioxidant activity decrease and possible short- and long-term consequences.
